# Competition improves robustness against loss of information

**DOI:** 10.3389/fncom.2015.00035

**Published:** 2015-03-25

**Authors:** Arash Kermani Kolankeh, Michael Teichmann, Fred H. Hamker

**Affiliations:** Department of Computer Science, Chemnitz University of TechnologyChemnitz, Germany

**Keywords:** competition, lateral inhibition, Hebbian learning, independent component analysis, non-negative matrix factorization, predictive coding/biased competition, occlusion, information loss

## Abstract

A substantial number of works have aimed at modeling the receptive field properties of the primary visual cortex (V1). Their evaluation criterion is usually the similarity of the model response properties to the recorded responses from biological organisms. However, as several algorithms were able to demonstrate some degree of similarity to biological data based on the existing criteria, we focus on the robustness against loss of information in the form of occlusions as an additional constraint for better understanding the algorithmic level of early vision in the brain. We try to investigate the influence of competition mechanisms on the robustness. Therefore, we compared four methods employing different competition mechanisms, namely, independent component analysis, non-negative matrix factorization with sparseness constraint, predictive coding/biased competition, and a Hebbian neural network with lateral inhibitory connections. Each of those methods is known to be capable of developing receptive fields comparable to those of V1 simple-cells. Since measuring the robustness of methods having simple-cell like receptive fields against occlusion is difficult, we measure the robustness using the classification accuracy on the MNIST hand written digit dataset. For this we trained all methods on the training set of the MNIST hand written digits dataset and tested them on a MNIST test set with different levels of occlusions. We observe that methods which employ competitive mechanisms have higher robustness against loss of information. Also the kind of the competition mechanisms plays an important role in robustness. Global feedback inhibition as employed in predictive coding/biased competition has an advantage compared to local lateral inhibition learned by an anti-Hebb rule.

## 1. Introduction

Several different learning approaches have been developed to model early vision, particularly at the level of V1 (Olshausen and Field, 1996; Bell and Sejnowski, 1997; Hoyer and Hyvärinen, 2000; Falconbridge et al., 2006; Rehn and Sommer, 2007; Wiltschut and Hamker, 2009; Spratling, 2010; Zylberberg et al., 2011). In many of the works, the proposed characteristics of the visual system have been considered as optimization objectives and thus as criteria for measuring the efficiency of coding. Several kinds of optimization objectives, like sparseness of activity (Olshausen and Field, 1996; Hoyer, 2004) or independence (Bell and Sejnowski, 1997; van Hateren and van der Schaaf, 1998) have been used for this purpose. One major criterion for evaluation of those models is their ability to develop oriented, band-pass receptive fields and the similarity of the distribution of receptive fields to observed ones in the macaque (Ringach, 2002). Although the match to biological data can be considered as one important criterion, further criteria are required to evaluate different approaches.

The visual system has the remarkable capability of robustness, or invariance, against different kinds of variances like, shift, rotation, scaling, occlusion, etc. of objects. This invariance is likely gradually achieved over different hierarchical levels, but robustness can be explained also in the form of information coding on the level of a single layer. This means, also units like V1 simple-cells show robustness against typical deformations of their preferred stimuli. In this work we have focused on the robustness under loss of information in the form of occlusion. Since typical forms of perturbations locally effecting V1-cells can be different lightning conditions—like reflections or flares; unclear media like soiled glasses, windows, heated air; or covered objects like the view through a fence—we define occlusion here as the random removal of visual information.

To investigate the role of different interactions, in fact competition, we compare four methods implementing different competition and learning strategies: Fast independent component analysis (FastICA) (Hyvärinen and Oja, 1997; Hoyer and Hyvärinen, 2000), non-negative matrix factorization with sparseness constraint (NMFSC) (Hoyer, 2004), predictive coding/biased competition (PC/BC) (Spratling, 2010), and a Hebbian neural network (further called HNN) with lateral inhibition based on Teichmann et al. (2012). Each method is capable of learning V1 simple-cell like receptive fields from natural images. FastICA was chosen as a method which tries to find new representations of data with minimal dependency between components without employing any kind of competition in the neural dynamics, but it enforces independent components via the learning rule. NMFSC uses a top-down, subtractive inhibition of the inputs to compute the outputs. NMFSC also keeps the output activity sparse on a desired, predefined level leading to unspecific competitive dynamics. PC/BC (Spratling, 2010) tries to find components minimizing the reconstruction error by a global error minimization employing inhibitory feedback connections. All of the above algorithms minimize a reconstruction error. While ICA minimizes a substractive reconstruction error, NMFSC (Hoyer, 2004) and PC/BC (Spratling, 2010) use divisive updating rules for the weight matrix that are derived from minimizing the Kullback-Leibler divergence (Lee and Seung, 1999). HNN uses Hebbian learning to learn the feedforward weights and anti-Hebbian learning to learn lateral inhibitory connections. The units compete via these lateral connections and suppress competing neurons locally based on the learned relations.

To evaluate the different algorithms trained all methods on the train set of the MNIST hand written digit dataset and measured their recognition accuracy on the occluded MNIST test set. The recognition accuracy was measured by feeding the activity patterns to a linear classifier. Here, the interesting aspect of each method was not its best accuracy in recognizing the classes, but its robustness in recognizing objects when the input was distorted, that is the change of the performance dependent on the level of occlusion.

## 2. Materials and methods

### 2.1. Dataset and preprocessing

We use the MNIST handwritten digit dataset^1^ to evaluate all methods. The dataset consists of 60,000 training images and 10,000 test images. All are centered, size normalized (28 × 28 pixel), and have black (i.e., zero) background. We downscale the images to 12 × 12 using the MATLAB (2013a) function imresize() by the factor of 0.40 with default parameters (i.e., bicubic interpolation). This matches the original configuration of the HNN input for learning V1 like receptive fields (Wiltschut and Hamker, 2009). In order to simulate the function of the early visual system up to the Lateral Geniculate Nucleus (LGN), which transfers signals from the eyes to V1, we whitened the images using the same method as in Olshausen and Field (1997). The whitened image contains positive and negative values. The positive part and the absolute values of the negative part of each whitened image were reshaped to vectors and concatenated to form a 288-dimensional input vector. The positive part resembles the on-center receptive fields of the Lateral Geniculate Nucleus (LGN) cells and the negative part the off-center receptive fields (Wiltschut and Hamker, 2009).

**Figure 1:**
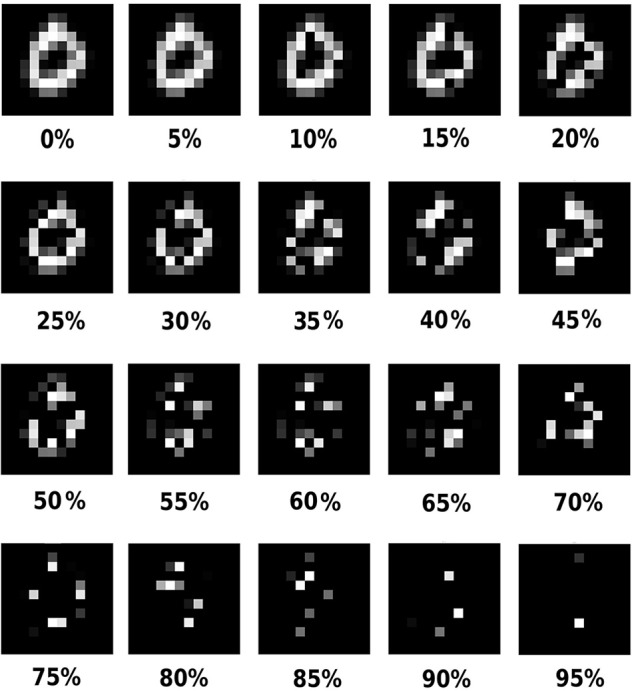
**An example of the input digits under 0-95% occlusions**.

We used a partially occluded test set to study the effect of loss of information on classification: the original non-occluded of MNIST and different occluded versions of it. A test set is formed by applying a particular occlusion level on all images in the original MNIST test set. That is, in each version, the level of occlusion was the same for all digits, although the position of the occluded pixels was generated randomly for each digit. The occluded test sets had an amount of 5–60%, in steps of 5%, occluded pixels. Only digit pixel and no background pixels were occluded. Occlusions were produced by randomly setting non-zero pixel values to zero before whitening an image (Figure 1). Since we are testing on all test sets we will further use the term “test set” to denote all of these test images. No occlusion was applied to the train set.

### 2.2. Models and training

In this section we will give a short introduction in the main principles and the training of the used methods. To facilitate comparison all methods are using 288 units. For our simulations we used software provided by the respective authors.

#### 2.2.1. Fast independent component analysis

In fast independent component analysis (FastICA; Hyvärinen and Oja, 1997), the goal is finding statistically independent components of the data by maximizing neg-entropy. Neg-entropy is a measure of non-gaussianity and non-gaussianity is in direct relation with independence; the more non-Gaussian the activity distributions, the more independent are the components. The problem can be stated as

x=Vy

or

y=Wx

where *V* is the mixing matrix and *W* its inverse, *x* is the input vector and *y* is the vector of sources or components which should be independent. ICA, as a generative method, tries to generate the inputs as a sum of components *y* weighted by the weights of the mixing matrix *V*. In FastICA matrices *V* and *W* are found in an optimization process which maximizes neg-entropy of the activities.

After *W* was determined on the (non-occluded) MNIST train set, we used *W* to calculate the output on the occluded test set by calculating *y*_*o*_ = *W* *x*_*o*_, where *x*_*o*_ stands for the occluded input and *y*_*o*_ for the corresponding output activities. Thus, the FastICA method has no competitive mechanism effecting the output, its just applying a linear transformation matrix on the input.

#### 2.2.2. Non-negative matrix factorization with sparseness constraint

In non-negative matrix factorization with sparseness constraint (NMFSC; Hoyer, 2004), the goal is to factorize the matrix of the input data in non-negative components and non-negative source matrices, imposing more biological plausibility in comparison to FastICA, as neuron responses are non-negative. NMFSC approaches the matrix of components *V* to satisfy *X* ≈ *V* ⊗ *Y*. Where *Y* is the matrix of output vectors and *X* the matrix of corresponding input vectors. *Y* and *V* are calculated while approaching the objective of reducing the difference between the input *X* and its reconstruction *V* ⊗ *Y*:

(1) $$V\leftarrow V⊗(XY^{T})\;⊘\;(VYY^{T})$$

Where ⊗ means element-wise multiplication and ⊘ element-wise division. One could say that the term (*XY*^*T*^) ⊘ (*VYY*^*T*^) is actually the modulated input which is used to update *V*. In some literature it is interpreted as a divisive form of feedback inhibition (Kompass, 2007; Spratling et al., 2009). This method, introduced by Lee and Seung (1999), tries to minimize the difference between the distributions of the input and its reconstruction based on Kullback-Leibler divergence.

In some other works this process is done by adding the subtractive difference between the input and its reconstruction to *V*. One could call both subtractive different and the divisive modulated input the inhibited input which is used for learning (Spratling et al., 2009).

The advantage of NMFSC to pure non-negative matrix factorization (NMF; Lee and Seung, 1999) is that the sparseness of the computed activities *Y* can be set to a desired level. An increase in the sparseness shifts the code from global to more local features (Hoyer, 2004). However, NMFSC deviates from a multiplicative update of the output *Y* and uses a subtractive one

$$Y\leftarrow Y-\mu V^{T}(VY-X)$$

Thus, the nodes compete with each other using a top-down, subtractive inhibition of their input. In order to obtain the desired level of sparseness a projection step is applied by keeping *V* fixed and looking for the closest *Y* which could both optimally cause to low reconstruction error and satisfy the sparseness constraint (for details see Hoyer, 2004, pp. 1462–1463). NMFSC also allows to control the sparseness of *V*, but this feature is not used by us.

**Figure 2:**
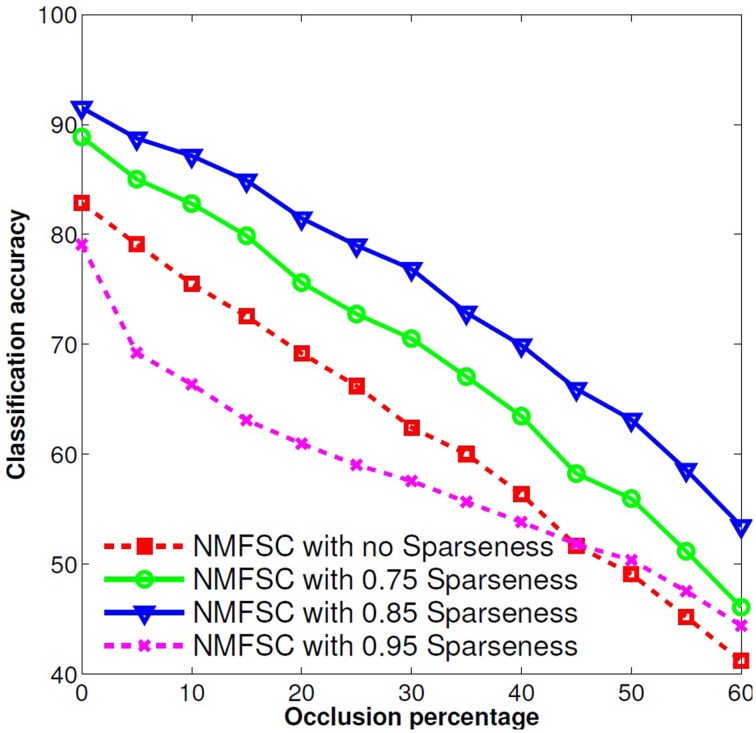
**Effect of different sparseness levels on the robustness of NMFSC, using the occluded MNIST test set**. A sparseness of 0.85 shows the best robustness. Very high or no sparseness reduces the performance.

To obtain the best classification accuracy, we tested four different sparseness levels (0, meaning no constraint; 0.75; 0.85; and 0.95). We found that 0.85 sparseness gives the best results (Figure 2). The same sparseness level was found by Hoyer (2004) as the best level to learn Gabor-like filters from natural images. Hoyer defines the sparseness level as the relation of the *L*_1_ norm to the *L*_2_ norm. Where a sparseness of zero denotes the densest output vector, this is when all outputs are equally active, and of one denotes the sparsest vector, when just one output is active. For equation and an illustration of different degrees of sparseness please see (Hoyer, 2004, pp. 1460–1461). After we have trained NMFSC on the train set, we used *V* to calculate the output on the occluded test set. For this, we kept the obtained *V* fixed and ran the optimization process for *Y*, approaching the predefined sparseness level for *Y* while trying to reduce the reconstruct error.

#### 2.2.3. Predictive coding/biased competition

In predictive coding/biased competition (PC/BC; Spratling, 2010), like in the two other generative models, the goal is finding components so that the output can resemble the input with minimal error. This method uses divisive input modulation (DIM), introduced in Spratling et al. (2009), which is in turn based on NMF. The modifications, in comparison to NMF, are mainly two. First, it is on-line, while NMF is a batch method. Second, in contrast to NMF which uses the component weight matrix both for computing the output and reconstructing the input, DIM considers two sets of weight matrices; feedforward for producing the output and feedback for producing the reconstruction of the input. The two weight matrices differ just in the form of normalization, which makes the method more powerful than NMF in the case of overlap and occlusion (Spratling et al., 2009). In PC/BC, the inputs are inhibited by being divided by their reconstruction. This is done explicitly in the units called error units. The error units basically do the same job as the term (*XY*^*T*^ ⊘ (*WYY*^*T*^) in (Equation 1) in NMF. Their activity is described as following:

$$e=x⊘(\epsilon_{1}+V^{T}y)$$

where *x* is the input vector, *y* is the output vector, *V* is the feedback weight matrix, and ϵ_1_ is a small value to avoid division by zero. The inhibited input from the error units is used for both producing the output and updating the weights. Thus, PC/BC uses in both cases a multiplicative updating, whereas NMFSC uses a subtractive one for the output.

To calculate the output the inhibited input is used:

$$y\leftarrow (\epsilon_{2}+y)\;⊗\;We$$

where ϵ_2_ is a random small number which prevents the output from being zero, *W* is the feedforward weight matrix and *e* is the activity vector of the error units. Based on the output activities *y* and the error units *e* the weights are adopted as following:

$$W\leftarrow W⊗\{(1+\beta y)[e^{T}-1]\}$$

where β is the learning rate. If the input and its reconstruction are equal, the error will be equal to unity and, thus, the weights will not change.

The input inhibition of PC/BC affects, besides the weight development, the output. Strong units suppressing weaker ones by removing their representation from the input. This is done in several iteration of updating the error units by the received reconstruction of the output units. This iterative process leads to a low reconstruction error and provides the competitive mechanism of PC/BC.

We trained PC/BC on 100,000 randomly, and potentially repeatedly, chosen digits from the 60,000 images of the MNIST train set and saved the weights for later calculating the outputs on the test set. Therefore, each image of the test set was presented for 200 iterations to the final network to achieve convergence of the outputs.

#### 2.2.4. The Hebbian neural network

Finally, we use a Hebbian neural network (HNN), employing the well accepted mechanisms of rate based threshold linear neurons and Hebbian learning. A set of neurons in one layer receive feedforward input and lateral inhibitory connections being the source of competition between the neurons. The connection strengths are learned using a Hebbian learning rule for the feedforward connections and an anti-Hebbian one for the lateral connections (Földiák, 1990; Wiltschut and Hamker, 2009; Teichmann et al., 2012). For simulation, we use a slightly modified version of the one previously published by Teichmann et al. (2012). To learn the feedforward weights, the model employs a set of different mechanisms like covariance learning with Oja normalization (Oja, 1982), regulated by an activity dependent homeostatic term (Teichmann et al., 2012). It uses calcium traces of the neuron activity instead of activities for learning. However, we use a fast trace so the model works similar to an activity based model (see Appendix for further model details).

**Figure 3:**
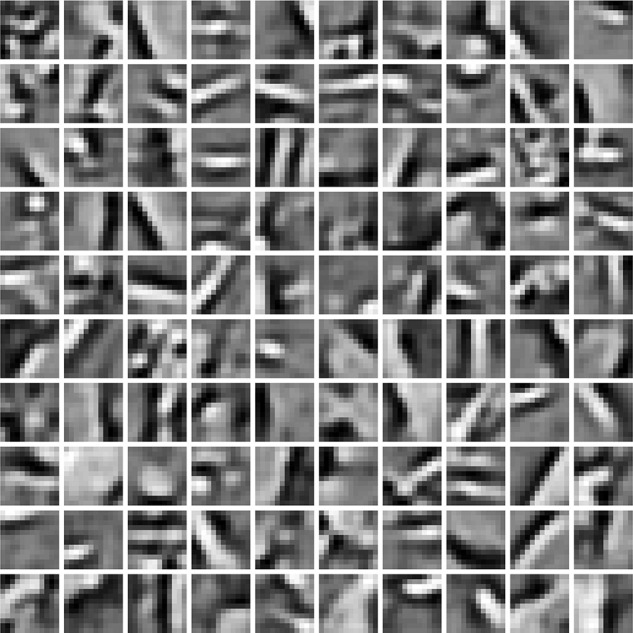
**Gabor-like receptive fields learned from natural images by the Hebbian neural network**.

Since Teichmann et al. (2012) demonstrated the model to learn V1 complex-cell properties we verified that the model used here, if trained on natural images, learns simple-cell receptive fields (Figure 3) to fulfill our main criteria for model selection.

In this kind of network, inhibitory lateral connections are the source of competition between units. During the learning process the lateral weights develop proportional to the correlated firing between units, leading to strong inhibition between units that are often coactive. Hence, units in the HNN tend to reduce coactivity in the training phase and thus build a sparse representation of the input. Consequently, each unit uses the stored knowledge in the lateral weights to suppress potentially competing units.

We trained the network on 200,000 randomly chosen digits from the train set. During training each image is been presented to the network for 100 time steps (ms) to allow for a convergence of the dynamics. After learning, we keep the weights fixed and use this network to obtain the responses on the images of the test set.

### 2.3. Classification

As a criterion for robustness, we considered the accuracy of a classifier on the top of each method. The idea behind was that the classifier would indicate by its performance drop to classify the digits if some information is lost. Thus, a method with a more stable representation should have less accuracy decrease under increasing levels of occlusion. We have decided to use a simple linear classifier as it is assumed that also the neural processing in the brain should facilitate linear classification (DiCarlo and Cox, 2007). To measure the accuracy of classification we use Linear Discriminant Analysis (LDA) on the output of the methods on the test set. That is, we used the MATLAB (2013a) function classify() with default parameters (linear discriminant function). The classifier is trained using the output of the respective method on the train set.

### 2.4. Visualization of weights and receptive fields

Obviously, if a method is able to learn a superior representation of the data, it will have a better robustness to the other ones. We visualize the weight matrices of all methods to get an insight into how the data are processed. If the methods share a similar character in their weight organization it can be assumed that this feedforward part of the processing shares similarities. Hence, the differences in the robustness of the methods have to come from the competitive mechanisms. Further, we can look at the receptive field shapes^2^ of the units, as the competition is typically not changing their overall shapes, indeed the inhibitory effects between units are considered.

Hence, we used two approaches for visualizing the receptive fields. One was representing the weight matrix of a unit as gray-scale images. As the weight matrices correspond to the on-center and off-center inputs, we subtract this two parts from each other (Wiltschut and Hamker, 2009). The strength of each of these weights was shown as the intensity of a pixel in the image, where white denotes the maximum weight, gray denotes zero, and black the minimum weight. As an alternative, to visualize the receptive fields, we used reverse correlation. In order to obtain the optimal stimulus of a unit, we weighted images containing 90 random dots in front of black (zero) background with their corresponding outputs from a single unit. The average of the result was shown as the receptive field. This way we could observe to which input parts each unit is sensitive, regarding the competition between the units. In other words the resulting matrices visualize the correlation between the input and output values of each unit.

## 3. Results

### 3.1. Learned receptive fields

**Figure 4:**
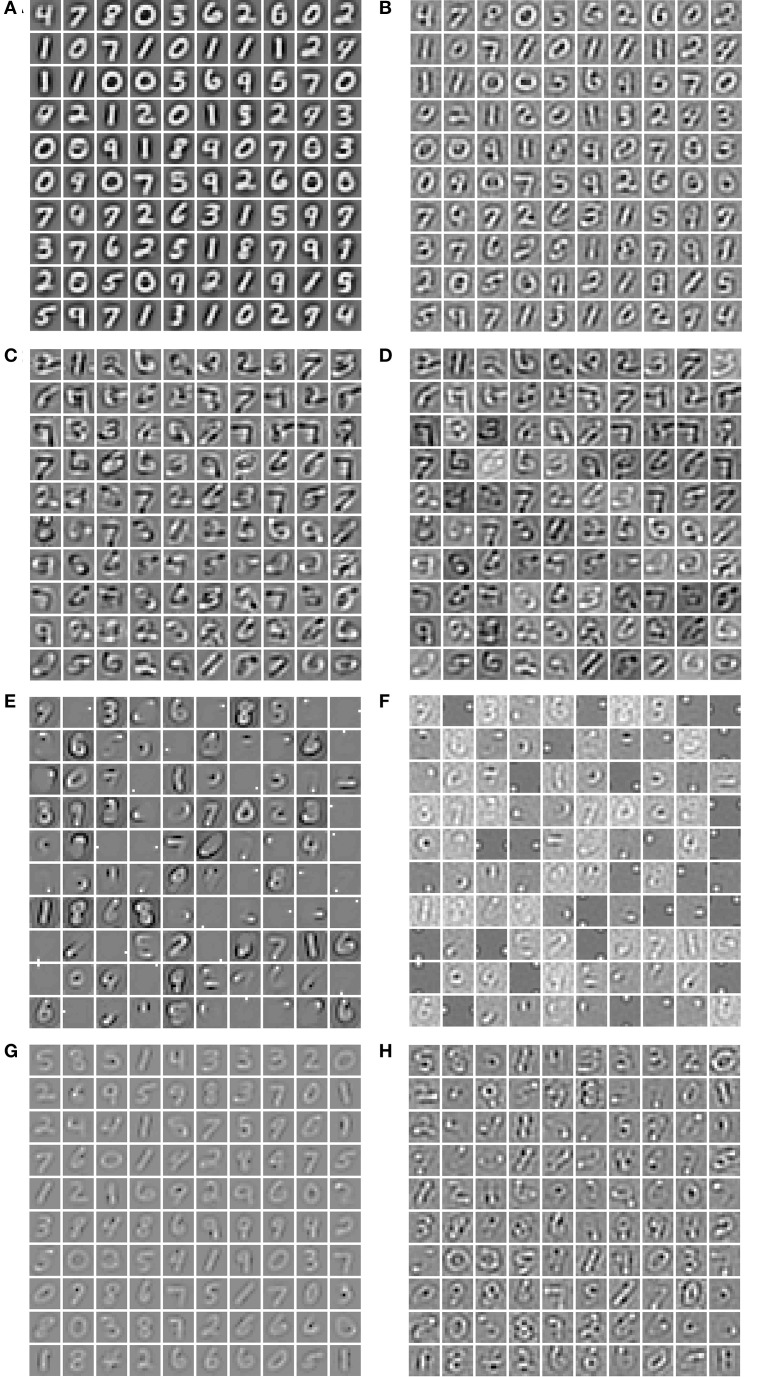
**Visualization of the feedforward weights and the receptive fields of 100 units, after training**. Off-weights where subtracted from on-weights and each plot is scaled so that white denotes the maximum value and black the minimum. **(A)** The feedforward weight matrices of the HNN and **(B)** its reverse correlation. **(C)** The component matrices of FastICA and **(D)** its reverse correlation. **(E)** The component matrices of NMFSC and **(F)** its reverse correlation. **(G)** The feedforward weights of PC/BC and **(H)** its reverse correlation.

In order to verify if the models represent the input data in a comparable way, we visualize the weight vectors and receptive fields of 100 units for each model (cf. Section 2.4). To visualize the weight vectors of the Hebbian neural network (HNN), we have used the feedforward weight matrices showing the driving stimulus of the neurons (Figure 4A). For FastICA, we visualize the mixing matrix *V* (Figure 4C). The *V* matrix of basis vectors is visualized for NMFSC (Figure 4E). In PC/BC, we show the feedforward matrices (Figure 4G). For each method we also show the receptive fields estimated by reverse correlation (Figures 4B,D,F,H), being not much different from the visualization of the weight matrices. All methods develop receptive fields with holistic forms of digits. Indeed, in NMFSC not all units show digit like shapes which may result from the chosen level of sparseness as mentioned in the methods.

### 3.2. Classification accuracy under occlusion

**Figure 5:**
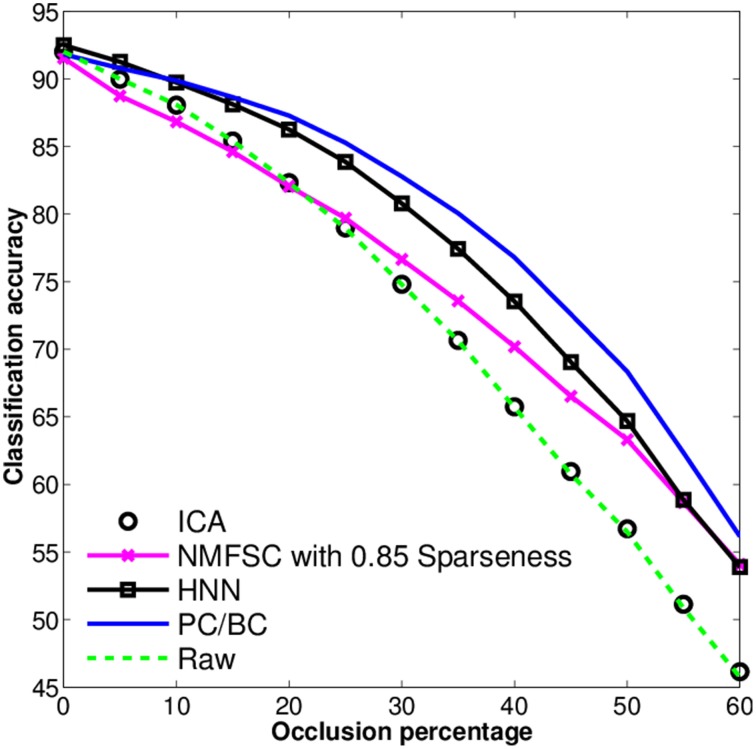
**Classification accuracy on the output of FastICA, NMFSC, HNN, and PC/BC, using the occluded MNIST test set**. Methods using competitive mechanisms show better robustness.

To investigate the differences in robustness to increasing levels of occlusions in the input, we have measured the classification accuracy of all methods and the raw data on the test set. We repeated the experiments 10 times with each algorithm under different starting conditions, i.e., randomly initialized weights. We do not show the error bars as they are zero for FastICA and NMFSC as they are deterministic and have been low for PC/BC and the HNN. We observed (Figure 5) that FastICA does not improve the classification accuracy to that of the raw data. NMFSC causes a super-linear decrease of classification accuracy with respect to the linear increase of occlusion. PC/BC shows the highest robustness against occlusion. The robustness of the HNN is higher than NMFSC and lower than PC/BC. The methods having more “advanced” competitive mechanisms perform better under increasing occlusions.

**Figure 6:**
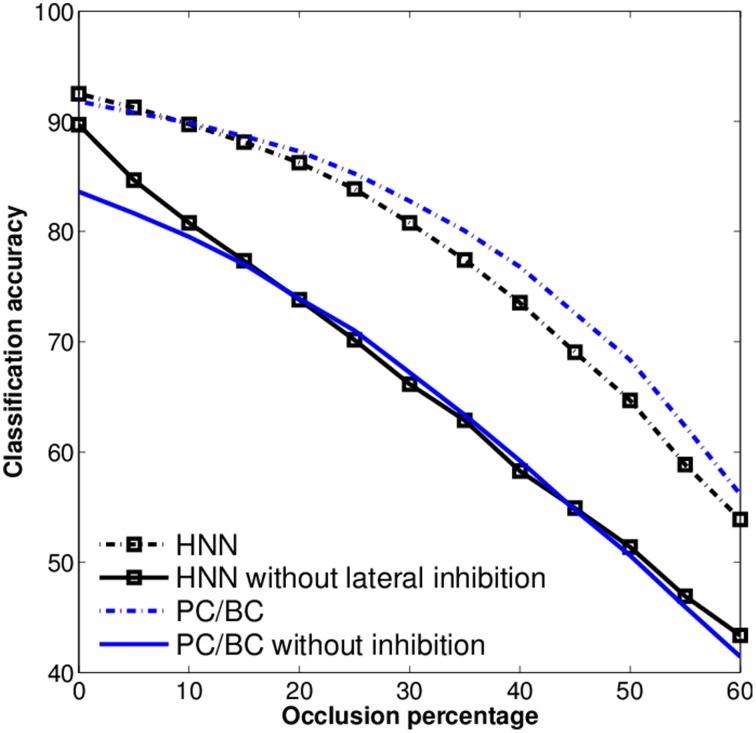
**Robustness of the HNN and PC/BC with and without inhibition, using the occluded MNIST test set**. Without the inhibitory connections the models show a sharp drop in performance against loss of information (occlusion).

To further investigate the influence of the competitive mechanisms we turn them off for PC/BC and the HNN. This is, setting the lateral inhibitory connections to zero for the HNN, and using only the first iteration step of PC/BC. The training of the classifier is repeated for these modified models. Both HNN and PC/BC will cause a very low performance even worse than the raw data when their competitive mechanisms are not used (Figure 6). Meaning that the competitive mechanism has a substantial influence on the accuracy under occlusion and the pure feedforward processing is not enough have robust recognition results.

### 3.3. Effect of occlusion on activity pattern

**Figure 7:**
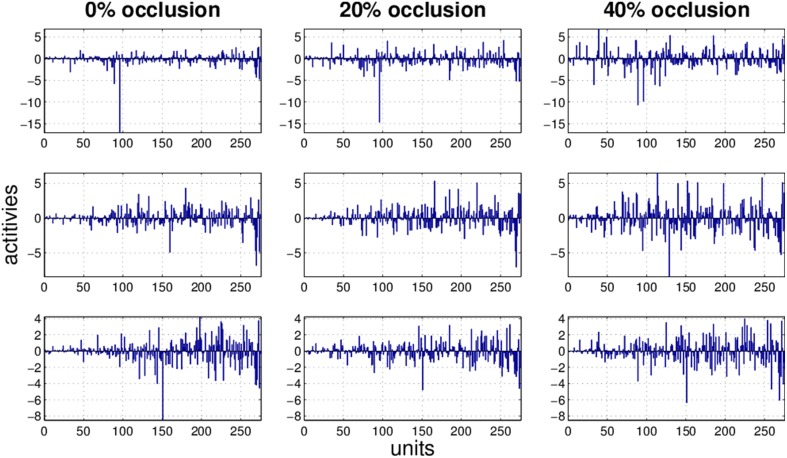
**Three examples (row) how the activity pattern vary, under 0, 20, and 40% of occlusion (column) in FastICA**.

**Figure 8:**
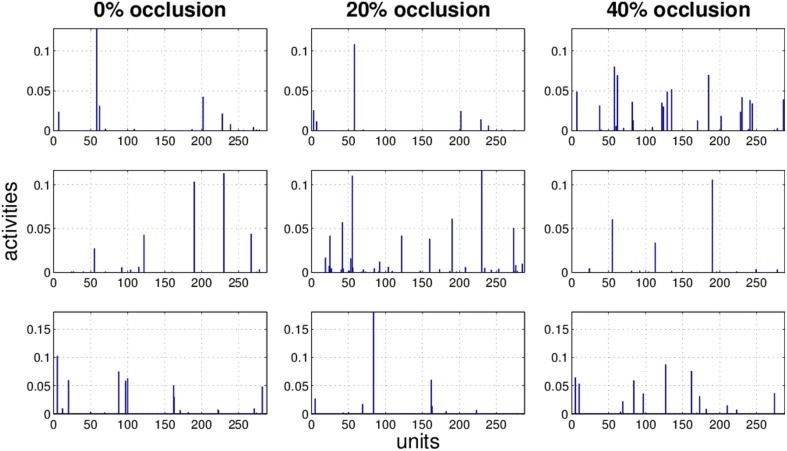
**Three examples (row) how the activity pattern varies, under 0, 20, and 40% of occlusion (column) in NMFSC**.

**Figure 9:**
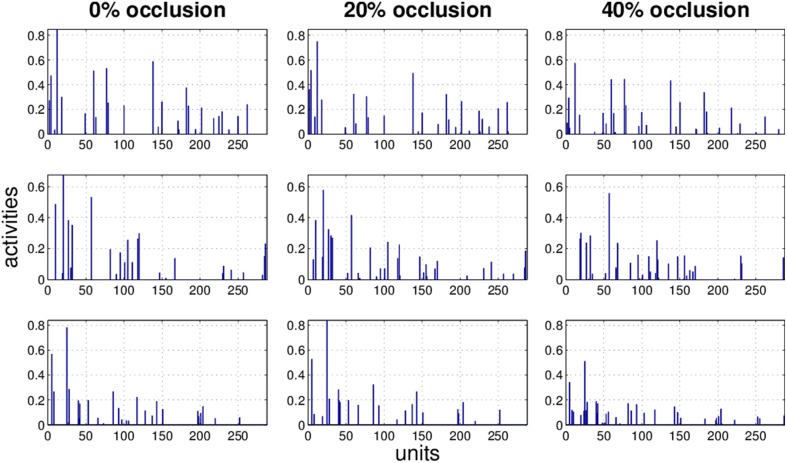
**Three examples (row) how the activity pattern varies, under 0, 20, and 40% of occlusion (column) in HNN**.

**Figure 10:**
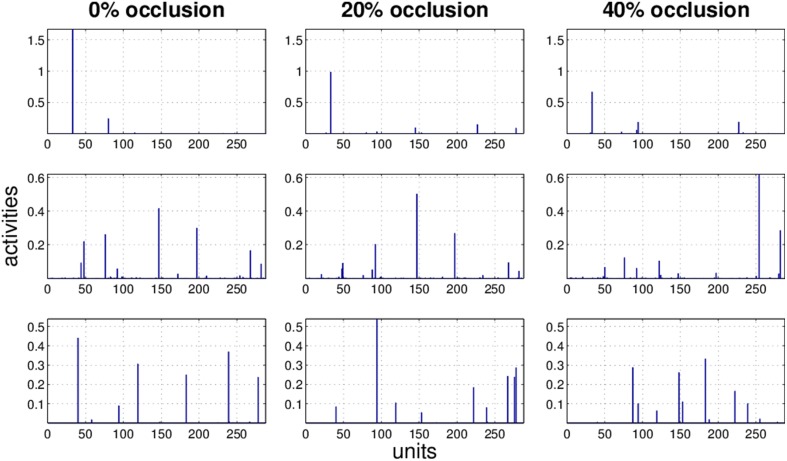
**Three examples (row) how the activity pattern varies, under 0, 20, and 40% of occlusion (column) in PC/BC**.

It is obvious that the activity pattern as a function of the input changes by increasing the occlusion in the input. The question is how stable the activity patterns of a method are when the occlusion in the input is increased. This is basically the same question as how much the classification accuracy is robust under loss of information. In Figures 7–10 the activity patterns corresponding to three random inputs under 0, 20, and 40% occlusion are illustrated. As one can see in NMFSC, HNN, and PC/BC the activity patterns corresponding to non-occluded input and low occluded (20%) are comparable. In FastICA, though, the activity patterns are not easily comparable as ICA by nature produces very dense activity patterns. The activity pattern of FastICA on the (non-occluded) train set have a mean sparseness (Hoyer, 2004) of 0.41, which is, in comparison with NMFSC with 0.89, HNN with 0.80, and PC/BC with 0.89 sparseness, quite dense. However, in all methods the activity pattern loses its original form when occlusion is increased.

To measure how stable the activity patterns of a method are, for different levels of occlusion, we used the cosine of the angle between the non-occluded and the occluded activity vector. We calculate the cosine on the test set with 20 and 40% occlusion (Table 1) and found that methods showing a more robust recognition accuracy also having a lesser turn in their activity vector. Exceptionally, the HNN shows a more stable code than PC/BC based on this measure.

**Table 1:** **Cosine between non-occluded and occluded activity patterns, calculated on the test set with having particular occlusion levels**.

	**20% Occlusion**	**40% Occlusion**
FastICA	0.65	0.46
NMFSC	0.71	0.61
PC/BC	0.78	0.61
HNN	0.87	0.76

A cosine of 1 denotes an equal direction and 0 denotes an orthogonal one. The stability of the activity patterns conform the results for the recognition accuracy, except the HNN shows a higher stability.

### 3.4. Selective inhibition in the Hebbian neural network

**Figure 11:**
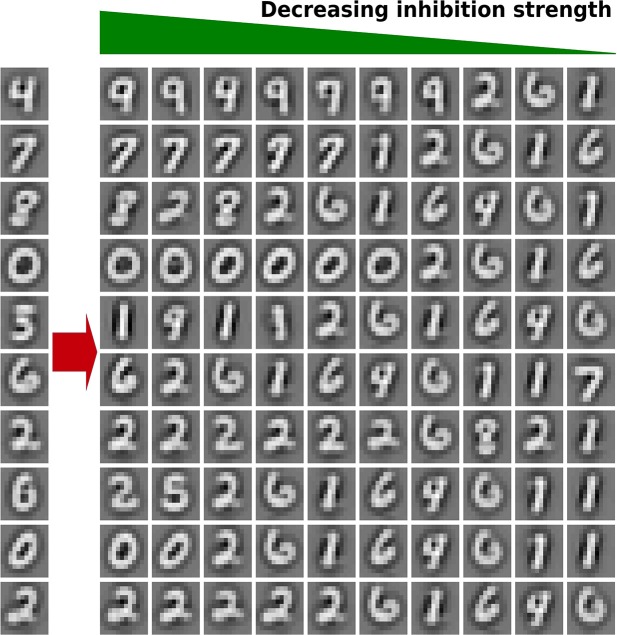
**Selective inhibition in the HNN**. On the left side the feedforward weights of 10 randomly chosen neurons are illustrated. Right of each neuron, the weights of 10 neurons receiving inhibition from this neuron are plotted, sorted from left to right by descending lateral weight strength (inhibition). The illustration shows that neurons having more similar feedforward weights are more inhibited than neurons having less similar weights.

To investigate the selectivity of inhibition in the HNN, we study the relation between the strength of the lateral connections and the similarity of the feedforward weights of a neuron to its laterally connected neurons by visualizing the feedforward weights of the laterally connected neurons sorted by the strength of the outgoing lateral connections. Therefore, we randomly select 10 neurons (left side) and plot the weights of the laterally connected neuron (Figure 11). As one can see, the shape of the feedforward weights of neurons being strongly inhibited are more similar to the weights of the inhibiting neuron than the ones which are lesser inhibited. This is, neurons have the strongest inhibition to neurons representing similar digits, mostly from the same class, followed by other classes sharing many similarities. Being expected as the strength of the inhibition is relative to the correlation of the neurons.

## 4. Discussion

We observed that the competitive mechanisms in the considered methods, FastICA, NMFSC, PC/BC, and HNN, have direct effect on their robustness under loss of information. Results showed that all methods have developed receptive fields similar to digit shapes and so the methods should be comparable. Apparently, this similarity itself cannot be used as a criterion for robustness against loss of information (occlusion). We observe that the receptive fields of FastICA are more similar to digits than the most of NMFSC, although, NMFSC shows a better accuracy under occlusion. However, without using its competition mechanism it behaves worser than FastICA. Further, HNN and PC/BC have the most clear receptive fields and the highest performances, indeed, without the competitive mechanism their accuracy drops lower than FastICA and NMFSC. Also the recognition accuracies of PC/BC and HNN with and without competition can not be explained by differences in the receptive field shapes. Without competition HNN behaves slightly better than PC/BC, whereas with competition PC/BC shows better accuracy. This means, the receptive field quality alone does not cause the observed higher robustness.

Without occlusions no method shows a strong superiority in the accuracy, indeed, they show clear differences when the input is distorted. Some models are more stable when the input is occluded. This stability is in line with the results of the classification accuracy. While the HNN shows the least change in the cosine between its population responses with and without occlusion, its classification accuracy is a bit weaker than the one of PC/BC for larger occlusions. The two dominant methods in this study, the HNN and PC/BC, employ different mechanisms for competition. These mechanisms help the systems to selectively inhibit the output of other units or respectively their input. In order to observe how much competition enhances robustness under occlusion, we have evaluated the classification performance when the competitive mechanisms were turned off. When the mechanisms are off, PC/BC and the HNN show a very low performance in the robustness to occlusions, as NMFSC without using the sparseness constraint. So obviously, the feedforward processing is not enough to obtain a sufficiently differentiated output and it can be assumed that competition is playing an essential role in the robustness of these systems.

We also observed that methods benefiting from a competitive mechanism are superior to FastICA, having no competitive mechanism on the output computation. FastICA linearly transfers the input space into a new space with least dependent components. When facing an image, FastICA produces a dense set of activities to describe the image in the new space. NMFSC without sparseness constraint acts as FastICA. However, when a reasonable level of sparseness is set for the activities of NMFSC it outperforms FastICA. The reason is that the sparseness constraint omits the appearance of redundant information to some extent. Indeed, a too sparse representation can remove some useful information and resulting in reduced accuracy. However, NMFSC acts weaker than the Hebbian neural network and PC/BC which may depend on the subtractive updating rule for output competition. Moreover, the optimal sparseness level is practically impossible, since a priori knowledge about the number features for an optimal representation is needed (Spratling, 2006). Also having this knowledge does not have to lead to an optimal result as different classes often need different amounts of features.

Among the three generative models FastICA, NMFSC, and PC/BC, PC/BC has been the superior model in this experiment. It uses a multiplicative updating rule to calculate the output activity. It finds the best matching units and removes their representations from the input of the other units, producing a sparse output while approaching a minimal reconstruction error. This online error minimization is realized by iteratively updating the error units representing the local elements of the reconstruction error and driving the output units. The HNN also has a competitive mechanism according to which the best matching units suppress other ones. In contrast to PC/BC, which tries to minimize the reconstruction error, the Hebbian Neural Network, as a whole, does not approach any explicit objective. It only exploits the knowledge from the training phase about the coactivity of units in order to suppress them. Thus, stronger units suppress potentially confusing weaker ones. That is, in HNN each unit is competing with other units based on its learned, local inhibitory weights, whereas PC/BC is actively using its distributed representation of the reconstruction error to minimize a global error signal. This may be the reason for the slight advantage of PC/BC against HNN for larger occlusions.

We conclude, that in order to achieve high robustness against loss of information in object recognition, one should focus on improving the competitive mechanism. Competition between units seems to play an important role in preventing the system from producing redundant activities. The experiments give also evidence that the cortical mechanisms of competition, as lateral inhibition, are the source of its robust recognition performance, even on single layer level. Similar effects to our V1 based evaluation can be found in deeper models of the visual cortex ventral stream, where even inhibitory lateral connections play an important role in robustness to occlusions (O'Reilly et al., 2013).

### Conflict of interest statement

The authors declare that the research was conducted in the absence of any commercial or financial relationships that could be construed as a potential conflict of interest.

## Footnotes

^1^http://yann.lecun.com/exdb/mnist/

^2^Receptive fields are here defined as a map of regions in the image where a unit is excited or inhibited if a stimulus is there (cf. Hubel and Wiesel, 1962).

## Appendix

### Computing neural activity in the HNN

As in Teichmann et al. (2012), the membrane potential *m*_*j*_ of a neuron *j* is calculated as the sum of each neuron's pre-synaptic input values *r*^*Input*^_*i*_ weighted by the corresponding synaptic strengths *w*_*ij*_. The resulting sum was decreased by the amount of inhibition from other layer neurons *r*_*k*_, weighted by the corresponding synaptic strengths of the lateral connections *c*_*kj*_ (Equation A1) and a non-linearity function *f* (Equation A2). In contrast to Teichmann et al. (2012), we add an additional decay term *r*_*j*_ which mimic the intrinsic adaption of the firing threshold (Turrigiano and Nelson, 2004) based on the temporal activity of the neuron (Equation A3). The resulting activation *r*_*j*_ of a neuron *j* is calculated using the top half rectified membrane potential (*m*_*j*_)^+^. Further, we apply a saturation term for high membrane potentials to avoid unrealistic high activations (Equation A4).

The change of the activity is described by

(A1) $$\tau_{m}\frac{\partial m_{j}}{\partial t}=\sum_{i}w_{ij}r_{i}^{Input}-\sum_{k,k\neq j}f\left(c_{kj}r_{k}\right)-{\bar{r}}_{j}-m_{j}$$

with τ_*m*_ = 10 and the non-linearity function

(A2) $$f(x)=d_{nl}\;\cdot \;\log\left(\frac{1+x}{1-x}\right)$$

and the temporal activity

(A3) $$\tau_{\bar{r}}\frac{\partial {\bar{r}}_{j}}{\partial t}=r_{j}-{\bar{r}}_{j}$$

with τ_*r*_ = 10000 and the transfer function

(A4) $$r_{j}=\{\begin{array}{ll} 0.5+\frac{1}{1+e^{-3.5(m_{j}-1)}} & \text{if}m_{j}>1\\ {\left(m_{j}\right)}^{+} & \text{else} \end{array}$$

### Changes in neural learning of the HNN

As in Teichmann et al. (2012), we use Oja's constraint (Oja, 1982) for normalizing the length of the weight vector, preventing an infinite increase of weights. In contrast to Oja, each neuron can have an individual weight vector length. Differently to our previous work, we calculate the factor α, determining the length, so that its change is based only on the squared membrane potential minus a fixed average membrane potential $\beta =\frac{1}{288}$. The value β is defined as 1 divided by amount of neurons in the layer (Equation A5).

(A5) $$\tau_{\alpha }\frac{\partial \alpha_{j}}{\partial t}=m_{j}^{2}-\beta$$

Since the learning rule was originally proposed for learning complex cells, the learning is based on calcium traces, following the activity of a neuron, to allow exploiting the temporal structure of the input. As this is not needed for learning simple cells or handwritten digits, we are using here a short time constant of τ_*Ca*_ = 10 for the calcium trace. The time constant is chosen that short to turn off the influence of previous stimuli on the learning result. Besides, we have shown in Teichmann et al. (2012) that using this time constant in a setup for learning complex cells, causes a huge amount of the cells with simple cell properties. All other parameters in this model are chosen as in Teichmann et al. (2012).
